# Evaluation of process parameters in shake flasks for mammalian cell culture

**DOI:** 10.1186/1753-6561-7-S6-P17

**Published:** 2013-12-04

**Authors:** Oscar B Platas, Volker Sandig, Ralf Pörtner, An-Ping Zeng

**Affiliations:** 1 Institute of Bioprocess and Biosystems Engineering, Hamburg University of Technology, Hamburg, D-21073, Germany; 2 ProBioGen AG, Berlin, D-13086, Germany

## Introduction

Shake flask cultivation is nowadays a routine technique during process development for mammalian cell lines. During shaken culture, changes in agitation velocity, shaking diameter or shake flask size affect the hydrodynamics in the shake flask. This might be reflected in the growth of the cultured cells.

Process parameters such as power input, mixing time, fluid velocity etc. have been determined and described mathematically for shake flasks used for microbial cultivation, but only to some extend for mammalian cell culture. Especially the relationship between these parameters and growth characteristics of mammalian cells is still a relatively uncovered issue.

In this work, process parameters like specific power input, mixing time, maximum fluid velocity and Reynolds number were determined for four different shake flasks (baffled and unbaffled) in a range of shaking velocities on a shaking machine. The specific growth rate (μ_max_) of the human industrial cell line AGE1.HN^® ^(ProBioGen AG, Berlin, Germany) was compared to the respective process parameters.

## Determination of process parameters

(1) **Power input (*P/V*) **was calculated according to experimental data, that have been published in correlations with the form of *Np = f(Re)*, where *Np *is the power number and *Re *the Reynolds number of the culture. The first correlation is based on the work by Büchs et al. [1,2], who used a modified *Np *analog to bioreactors, and fited the experimental *Np' *data to *Re*. The second correlation used is based on the work of Kato et al. [3]. Here, the calculation of the Reynolds number considers the diameter of the shaker (*d_o_*) instead of the inner flask diameter (*d_i_*).

(2) **Mixing time (Θ_95_) **was determined by means of the decolourization method (I/KI titrated with Na_2_S_2_O_3_). Decolourization time course was video recorded and visually analyzed.

(3) **Maximum fluid velocity (*u_i_*) **was calculated at the maximum flask's inner diameter.

(4) **Reynolds number (Re) **was calculated as *Re *= *ρNd^2^/η*, with *d *= *d_i_*, and *d = d_o_*, for the methods published by Büchs et al., and Kato el al. respectively.

A modified di (*d_i,mod_*) was used for calculations of parameters in baffled flasks. This number considers the flask's depth into the flask circumference. The **average specific growth rate μ_max _**was employed as indicator for growth performance.

## Relationship between cell growth and process transfer criteria

**Figure 1:**
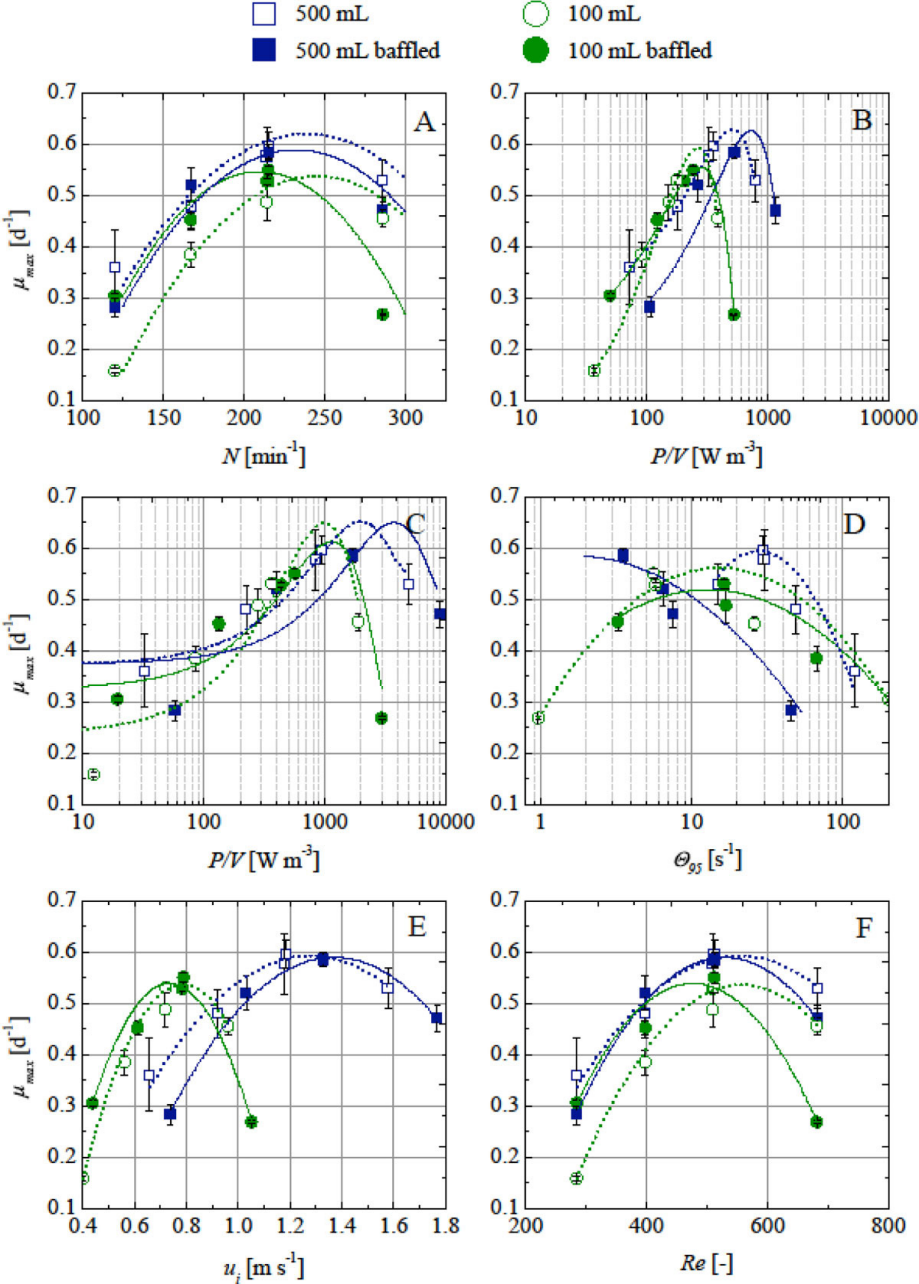
**Relationship between maximum specific growth rate *μ_max _*and the process parameters in shake flask culture**. **A) **Shaking velocity, **B) **Power input calculated with the method by Büchs et al., **C) **Power input calculated by the method by Kato el al., **D) **Mixing time, **E) **Maximum fluid velocity, F) Reynolds number with *d *= *d_o_*.

Figure 1 shows the dependency of the **average specific growth rate μ_max _**of AGE1.HN^® ^cells on the process parameters of the cultures performed in shake flasks. A shaking velocity of 200-250 min^-1 ^seems to be optimal for the cell growth rate. A maximal specific growth rate was observed in a close range of power input at 200-400 W m^-3 ^according to the method of Büchs et al. and at 400-1000 W m^-3 ^for the method of Kato et al. used for Re calculation. As has been shown for the culture of AGE1.HN^® ^cells in bench-top bioreactors [4], a range of mixing time values between 8 and 13 seconds can be identified here as common for all shake flasks too. The process operational windows identified in this work can lead to a significant reduction in the growth differences of mammalian cells in the context of standardization and reproducibility of shake flask cultures.

## Conclusions

Our results point to regions of the studied parameters, where common operation windows can be identified for μ_max_. In these process windows the cells show a similar μ_max _in different shake flask, making cell growth comparable. These process windows are common for the flasks, independently of their size and the number of baffles.

The data obtained in this work can be used for process standardization and comparability of results obtained in shaken systems i.e. to guarantee consistency of results generated during laboratory studies with mammalian cells.
